# Conservation of kinase-phosphorylation site pairings: Evidence for an evolutionarily dynamic phosphoproteome

**DOI:** 10.1371/journal.pone.0202036

**Published:** 2018-08-14

**Authors:** Megan McDonald, Brett Trost, Scott Napper

**Affiliations:** 1 Vaccine and Infectious Disease Organization-International Vaccine Research Center, University of Saskatchewan, Saskatoon, Saskatchewan, Canada; 2 Department of Biochemistry, University of Saskatchewan, Saskatoon, Saskatchewan, Canada; 3 Department of Computer Science, University of Saskatchewan, Saskatoon, Saskatchewan, Canada; University of Georgia, UNITED STATES

## Abstract

Kinase-mediated protein phosphorylation is a central mechanism for regulation of cellular responses and phenotypes. While considerable information is available regarding the evolutionary relationships within the kinase family, as well as the evolutionary conservation of phosphorylation sites, each aspect of this partnership is typically considered in isolation, despite their clear functional relationship. Here, to offer a more holistic perspective on the evolution of protein phosphorylation, the conservation of protein phosphorylation sites is considered in the context of the conservation of the corresponding modifying kinases. Specifically, conservation of defined kinase-phosphorylation site pairings (KPSPs), as well as of each of the component parts (the kinase and the phosphorylation site), were examined across a range of species. As expected, greater evolutionary distance between species was generally associated with lower probability of KPSP conservation, and only a small fraction of KPSPs were maintained across all species, with the vast majority of KPSP losses due to the absence of the phosphorylation site. This supports a model in which a relatively stable kinome promotes the emergence of functional substrates from an evolutionarily malleable phosphoproteome.

## Introduction

In terms of frequency and functional consequences, kinase-mediated phosphorylation is arguably the most important post-translational modification of proteins. For example, in human, 518 protein kinases catalyze an estimated 100,000 phosphorylation events [1, 2]. That such a significant portion of the proteome is modified via phosphorylation, often with pivotal roles in the regulation of protein function and subsequent phenotypes, supports the notion that changes in patterns of kinase-mediated protein phosphorylation have the potential to contribute substantially to evolutionary phenotypic variation, and, more specifically, to the molecular diversity from which organisms evolve [3].

**Fig 1:**
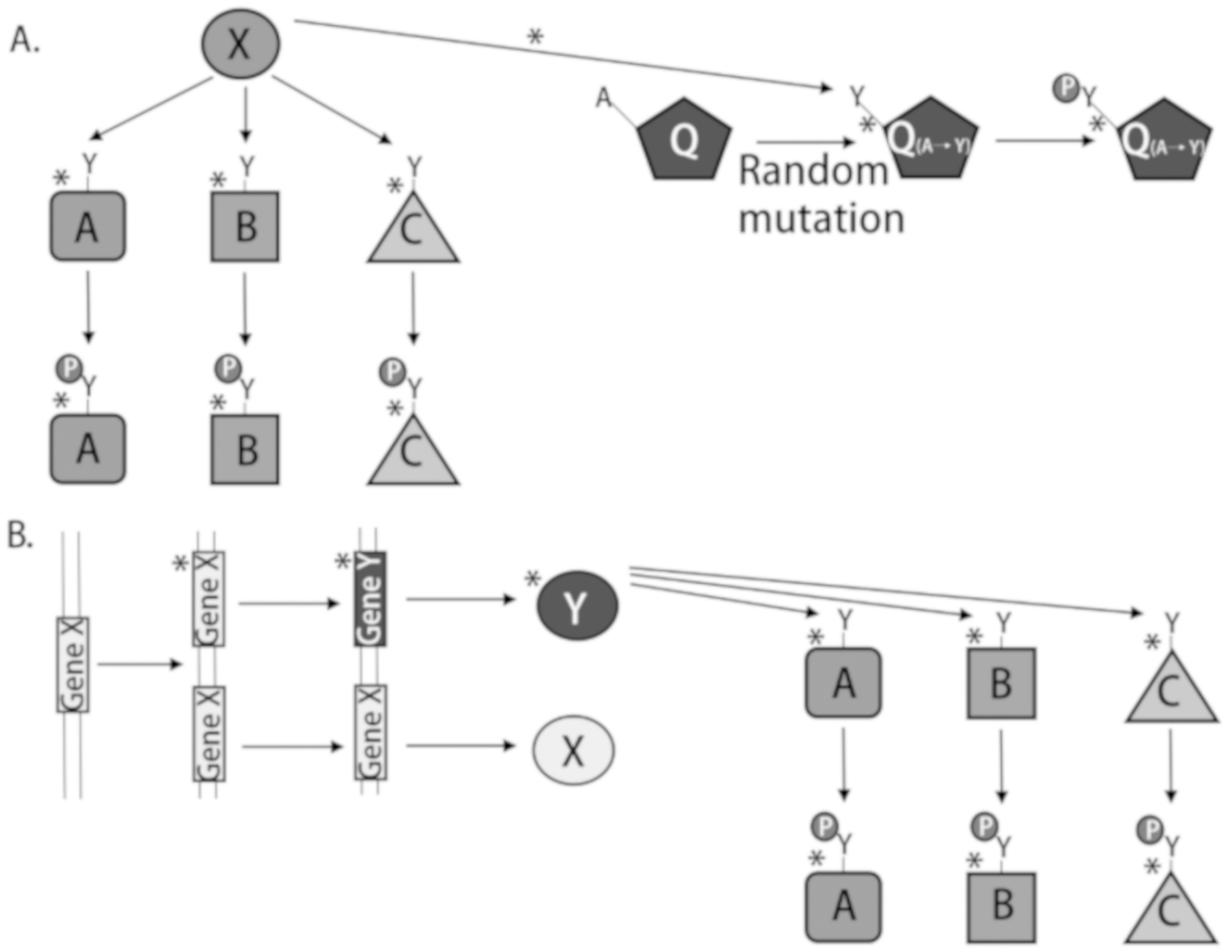
Proposed models for establishment of kinase-phosphorylation site pairing. (A) Pre-existing kinase X exists to perform some function in the cell, such as phosphorylation of substrates A, B, and C. By random mutation, a new phosphosite is generated on protein Q (in this case, A→Y). Protein X is able to phosphorylate the new site on Q and this confers a new level of function and/or regulation for Q. (B) Potential phosphosites exist in proteins A, B, and C. Gene X produces a protein X that cannot phosphorylate the sites on A, B, and C. Through gene duplication, a second gene X is generated in the genome and through subsequent random mutation events the second gene diverges from the sequence of the original gene X over time, creating a new gene Y. Gene Y produces protein kinase Y that is able to phosphorylate the sites already existing on A, B, and C, thus regulating and/or modulating them and their function(s) through phosphorylation.

From an evolutionary perspective, there are two basic models for the establishment of a kinase-phosphorylation site pairing (KPSP). Importantly, a KPSP, as defined here, relates only to the ability of a kinase to modify a substrate site, independent of any functional consequences of that modification. In one scenario, pre-existing kinases modify new substrate phosphoacceptor sites that are generated within the proteome through mutations (Fig 1A). In this model, kinase activity represents a constant against which phosphorylation sites emerge from a dynamic proteome. Phosphorylation events that bestow a phenotypic advantage are selected for over time. In a second model, phosphorylatable sites pre-date the kinases that modify them (Fig 1B). Here, through gene duplication and genetic drift, new kinases emerge to phosphorylate pre-existing phosphorylation sites. Phenotypic advantages as a consequence of these modifications serve to select the newfound kinase. While these models are likely not mutually exclusive, one may be favoured. Here, we present a preliminary investigation of these models by determining the extent of KPSP conservation in a set of organisms with widely varying evolutionary distance from human. Consideration of the conservation of kinases and substrates as functional pairings, rather than as distinct entities, may provide insight into the evolutionary mechanisms driving KPSPs.

## Materials and methods

A modified version of DAPPLE [4] was used to predict phosphorylation sites in several organisms based on sequence homology to experimentally-determined human phosphorylation sites. DAPPLE’s input consists of the proteome of the target organism and a database of known phosphorylation sites. Specifically, known phosphorylation sites are represented as short peptides (15-mers) with the phosphorylated residue in the centre. BLAST searches are performed using these peptides as queries and the proteome of the target organism as the database. DAPPLE’s output contains several types of information regarding each query peptide and its best match in the target proteome, most importantly the number of sequence differences between them [4]. The fewer sequence differences, the more likely the matching peptide represents a real phosphorylation site [5]. For this study, DAPPLE was altered to utilize the kinase/substrate dataset from PhosphoSitePlus (Kinase_Substrate_Dataset.gz, downloaded Mar. 3, 2015), which includes the kinase known to phosphorylate a given site (i.e., known KPSPs) [6, 7]. Specifically, each entry contains the UniProt accession number of the protein containing a given phosphorylation site, the position of the phosphorylation site, and the UniProt accession number of the kinase catalyzing the phosphorylation reaction. The dataset was checked for redundant KPSPs, and none were found. The database was then filtered to include only KPSPs from human. Most phosphorylation sites in the dataset were phosphorylated by only one kinase; for those that were phosphorylated by more than one, for simplicity only the first KPSP listed was retained. Finally, KPSPs for which the accession number of either the kinase or the target protein was not found in the UniProt human proteome were removed. The final, filtered dataset included a total of 5,915 known KPSPs.

The modified version of DAPPLE, combined with this filtered database, was used to determine the conservation of KPSPs between humans and *Macaca mulatta* (rhesus macaque), *Mus musculus* (mouse), *Gallus gallus* (chicken), *Danio rerio* (zebrafish), *Drosophila melanogaster* (fruit fly), *Caenorhabditis elegans* (nematode worm), *Saccharomyces* *cerevisiae* (yeast), and *Dictyostelium discoideum* (slime mold). For each known human KPSP in the PhosphoSitePlus kinase/substrate database and each of these organisms, we determined whether or not the phosphorylation site and kinase were conserved between human and that organism. A phosphorylation site was considered conserved if it satisfied the following criteria. First, there were fewer than seven sequence differences between the query peptide and its best hit in the proteome of the target organism (for a justification of this number, see [5]). Second, fewer than five of those sequence differences were non-conservative substitutions. Third, the location of the phosphorylated residue in the full protein corresponding to the query peptide was close to its position in the full protein corresponding to the hit peptide. More precisely, let P_Q_ denote the position of the phosphorylated residue in the query protein and P_H_ denote the position of the (putative) phosphorylated residue in the hit protein. Also, let L_Q_ denote the length of the query protein. To satisfy this criterion, we required that P_H_ be greater than P_Q_—0.2*L_Q_ and less than P_Q_ + 0.2*L_Q_. Fourth, the residue in the hit peptide that corresponded to the phosphorylated residue in the query peptide was either one of the canonical phosphorylatable residues (i.e., Ser, Thr, or Tyr) or matched the phosphorylated residue in the query peptide (i.e., if the query and hit phosphorylated residue were the same non-canonical phosphorylatable residue, such as His). If any of these four criteria were not met, then the phosphorylation site was not considered conserved in that organism. To determine if the kinase was conserved, the sequence of that kinase was retrieved from UniProt, and the reciprocal BLAST hits method (RBH) was used to determine whether an orthologue of that kinase exists the target organism [4]. We chose RBH as our primary method of orthology detection because it is commonly used and well understood and because it most closely parallels the procedure used to detect phosphorylation site conservation. However, for comparison purposes, we also analyzed our data using two more sophisticated methods for orthology detection: Hieranoid, a graph-based method [8,9], and DIOPT, an ensemble-based method [10]. For Hieranoid, a human kinase with UniProt accession number X was considered to have an orthologue in a given species if there was an entry containing X and a protein from that species in the file http://hieranoid.sbc.su.se/download/H2/Hieranoid2-Pairs.gz. For DIOPT, a human kinase was considered to have an orthologue in a given species if the “rank” field was “high”. DIOPT did not contain predictions for three of the organisms examined in this study (rhesus macaque, chicken, and slime mold).

**Fig 2:**
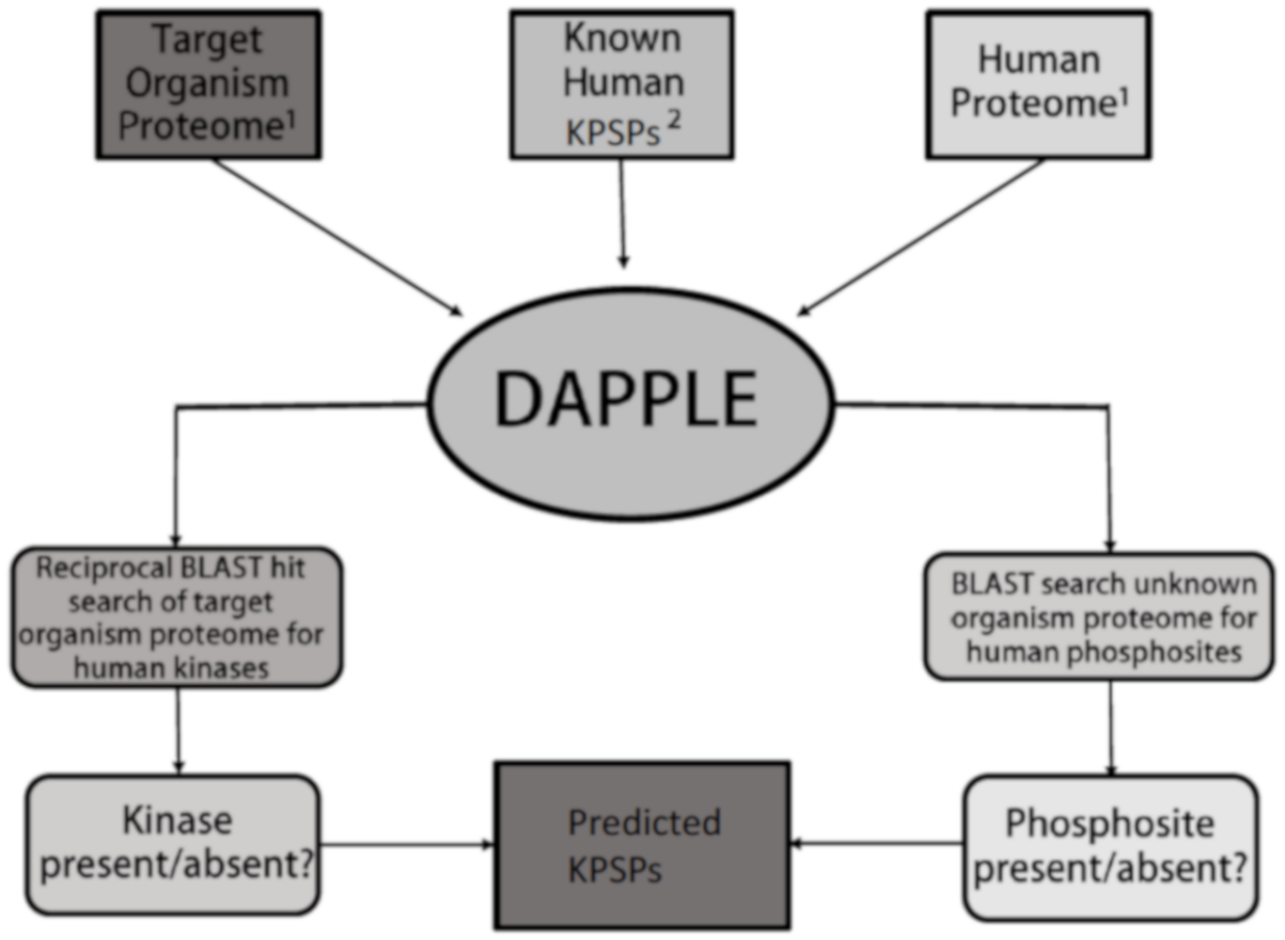
Methodology flowchart. Information input from databases of the target organism proteome, the human proteome, and known human KPSPs are integrated by DAPPLE to output information on the presence/absence of kinases, phosphosites, and/or KSPPs. 1 UniProt Proteomic Database. 2 PhosphoSitePlus Kinase-Substrate Database [human entries] (http://www.phosphosite.org/downloads/Kinase_Substrate_Dataset.gz).

The procedures for detecting both kinase conservation and phosphorylation site conservation ignored isoforms; for example, if protein P12345-3 in human was searched against the chicken proteome and its best match was P67890-2, and the best match when P67890-2 was searched against the human proteome was P12345-4, then P12345 and P67890 were considered to be reciprocal best BLAST hits. A flowchart depicting the methodology used to ascertain the conservation of KPSPs is given (Fig 2).

To investigate whether phosphorylation sites with known regulatory functions were more likely to be conserved, we downloaded the file containing known regulatory sites from the PhosphoSitePlus database (Regulatory_sites.gz, downloaded Mar. 16, 2018), and annotated each of our 5,915 known human KPSPs as to whether or not the phosphorylation site component of the KPSP had a known regulatory role.

## Results

Prior to comparing the conservation of phosphorylation sites and their cognate kinases, we sought to determine whether the criteria described above for assessing phosphorylation site conservation (fewer than seven substitutions between the query sequence and the hit sequence, fewer than five non-conservative substitutions, etc.) were erroneously rejecting experimentally-determined phosphorylation sites. The PhosphoSitePlus kinase-substrate database contained experimentally-determined phosphorylation sites for two of the non-human organisms analyzed in this study: chicken and mouse. For each of these organisms, we identified DAPPLE hits (from which human phosphorylation sites were used as queries) that matched a known site in that organism. From these, we calculated the proportion that were rejected (i.e., the phosphorylation site was deemed to be non-conserved) based on the criteria above. Reassuringly, we observed a very low rate of false rejection: of 449 DAPPLE hits in mouse that matched a known mouse site, just nine (2%) failed the aforementioned criteria (S1 File). For chicken, 2/15 (13.3%) DAPPLE hits failed our criteria (S1 File). The higher percentage for chicken could reflect the greater evolutionary distance between human and chicken compared to human and mouse, or could simply be due to the small number of known chicken phosphorylation sites. While these results give some confidence to our conservation criteria, it should be noted that this analysis has the limitation that the more distantly-related organisms from human that we analyzed in this study are not represented because of the lack of known phosphorylation sites.

**Fig 3:**
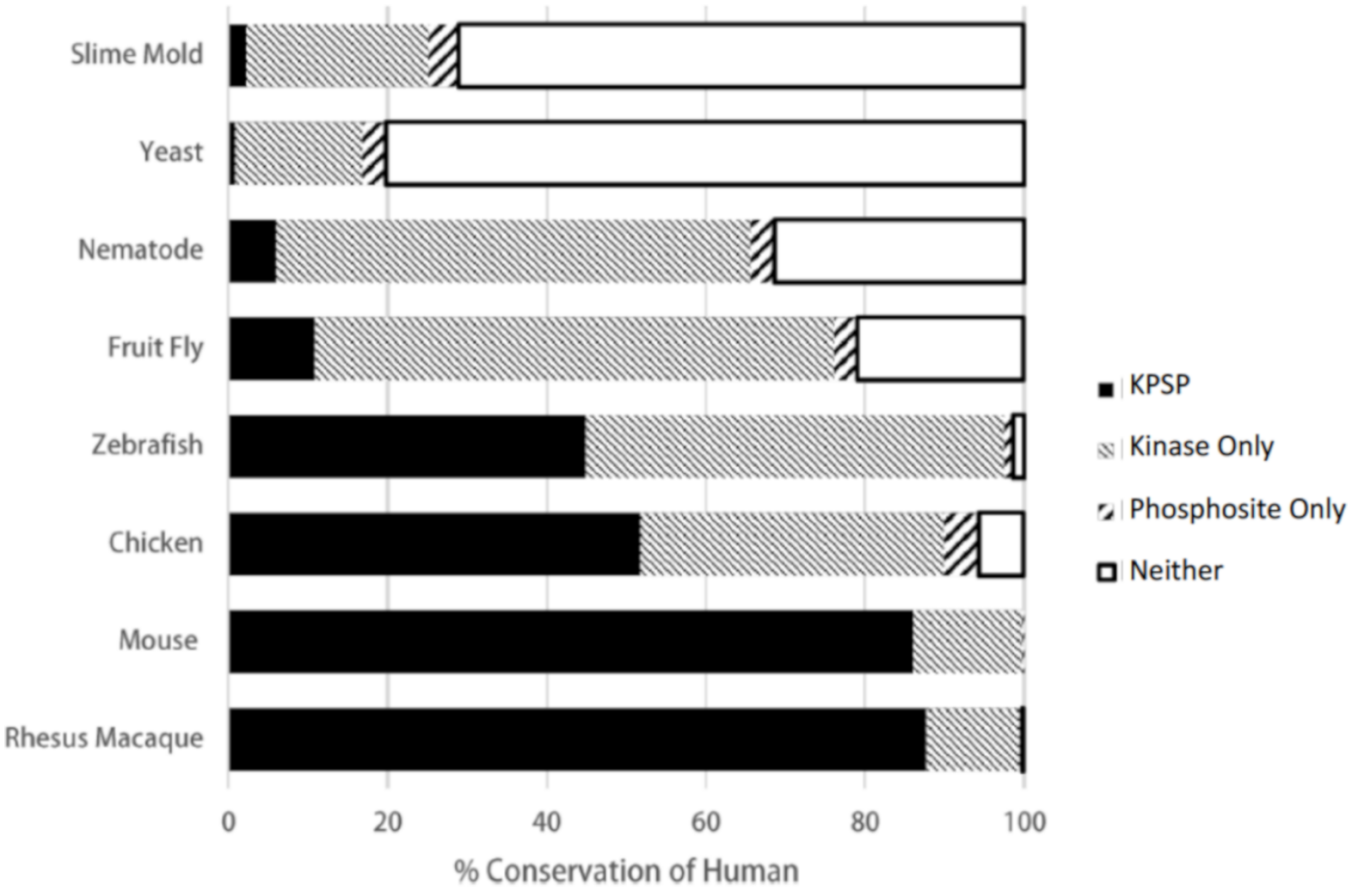
Percent conservation of human kinase-phosphorylation site pairings. The percent of conserved human KPSPs, phosphosites only (kinase absent), and kinases only (phosphosites absence) predicted by DAPPLE in an array of organisms ranging from early eukaryotic organisms to primates.

**Fig 4:**
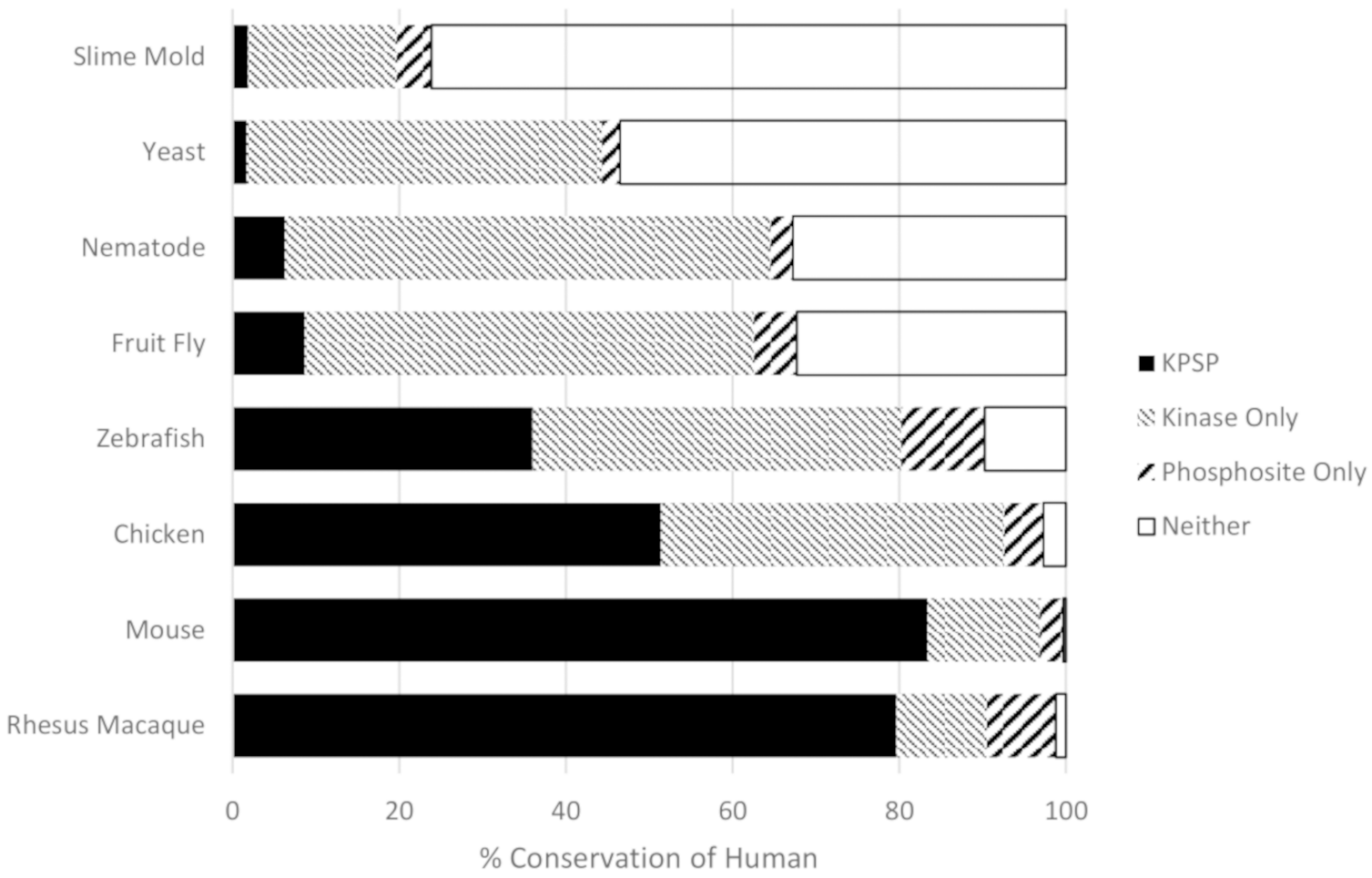
Percent conservation of human kinase-phosphorylation site pairings predicted by Hieranoid. The percent of conserved human KPSPs, phosphosites only (kinase absent), and kinases only (phosphosites absence) predicted using Hieranoid to detect orthologs in an array of organisms ranging from early eukaryotic organisms to primates.

**Fig 5:**
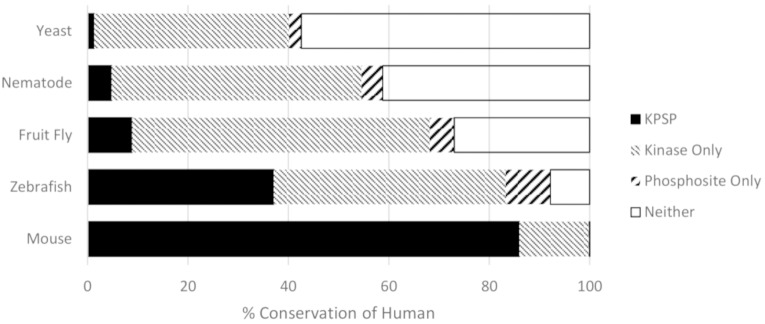
Percent conservation of human kinase-phosphorylation site pairings predicted by DIOPT. The percent of conserved human KPSPs, phosphosites only (kinase absent), and kinases only (phosphosites absence) predicted using DIOPT to detect orthologs in an array of organisms ranging from early eukaryotic organisms to mouse.

Once we were satisfied with our criteria for ascertaining phosphorylation site conservation, we determined conservation of human phosphorylation sites and kinases in rhesus macaque, mouse, chicken, zebrafish, fruit fly, nematode worm, yeast, and slime mold. For each known human KPSP and each organism, we determined whether: a) both the kinase and the phosphorylation site were conserved; b) only the phosphorylation site was conserved; c) only the kinase was conserved; or d) neither was conserved (Fig 3). We also confirmed our method of choice in orthology detection and KPSP prediction (RBH) by using two alternative methods, Hieranoid and DIOPT, for comparison (Figs 4 and 5). Similar trends in percent of conserved human KPSPs, phosphosite only, kinase only, and neither conserved can be seen using our chosen method of orthology detection (RBH) and both alternative methods. The extent to which functional pairings of kinases and their substrates were maintained across species reflected the evolutionary distance between these species, as there was a clear relationship between the evolutionary distance and the probability of maintaining a KPSP. That only a small fraction (less than 1%) of the KPSPs were maintained across all of the species considered indicated that while kinase-mediated phosphorylation is a common mechanism in all eukaryotes, specific phosphorylation events vary considerably across species. Even between closely-related mammalian species, such as monkey and human, a considerable fraction (approximately 15%) of the pairings were not maintained. Naturally, the extent to which phosphorylation events and kinases will be predicted to be conserved depends on the stringency of the criteria employed. However, the priority of this work was to identify relative trends rather than absolute values, and the same criteria were consistently applied to all species. Finally, other researchers, using independent approaches, have reported similar levels of conservation of phosphorylation sites, giving confidence to our findings; for example, a previous comparison of human and mouse orthologues found that 12% of phosphoserine sites and 15% of phosphothreonine sites were not conserved [11]. S2 File contains detailed information on the conservation of the 5,915 known KPSPs in each of the species considered.

The unmaintained KPSPs can be sub-divided into three groups: a) absence of the kinase only, b) absence of the phosphorylation site only, or c) absence of both the kinase and phosphorylation site. With increasing evolutionary distance, there was a trend toward the eventual complete elimination of both aspects of the KPSP; however, in instances where only the kinase or only the phosphorylation site was missing, the vast majority reflect absence of the substrate. This may suggest that for the majority of these instances, the existence of the kinase preceded the establishment of the phosphorylation site.

Finally, we assessed whether sites with known regulatory roles were more likely to be conserved. In general, human sites that were conserved in a given organism were more likely to have regulatory roles than non-conserved sites (Table 1, S2 File). Interestingly, this relationship was strongest for organisms with moderate evolutionary distance to human, and surprisingly was absent from the most closely related organism to human (rhesus macaque). We hypothesized that these patterns may relate to noise in the regulatory role data—specifically, that some of the regulatory sites with little/weak evidence (e.g., only high-throughput assays) may be false positives. Thus, we re-analyzed the data using only those sites for which evidence for a regulatory role was provided by at least two low-throughput references. The resulting data support a clearer difference between the “both conserved” and “only kinase conserved” categories for rhesus macaque, and also a more consistent pattern across all organisms examined (Table 1, S2 File).

**Table 1:** Proportion of human phosphorylation sites in each category annotated as having a known regulatory function in PhosphoSitePlus using reciprocal blast.

Organism	Both conserved	Only kinase conserved	Only phosphosite conserved	Neither conserved
All regulatory sites in PhosphoSitePlus				
Rhesus macaque	3046/5192 (58.6%)	399/698 (57.1%)	11/12 (91.6%)	13/13 (100.0%)
Mouse	3058/5090 (60.0%)	403/816 (49.3%)	8/9 (88.8%)	0/0 (N/A)
Chicken	1951/3059 (63.7%)	1327/2260 (58.7%)	92/263 (34.9%)	99/333 (29.7%)
Zebrafish	1704/2658 (64.1%)	1692/3111 (54.3%)	34/67 (50.7%)	39/79 (49.3%)
Fruit fly	432/641 (67.3%)	2375/3862 (61.4%)	92/175 (52.5%)	570/1237 (46.0%)
Nematode	224/355 (63.0%)	2180/3525 (61.8%)	87/177 (49.1%)	978/1858 (52.6%)
Yeast	25/47 (53.1%)	496/946 (52.4%)	107/180 (59.4%)	2841/4742 (59.9%)
Slime mold	84/133 (63.1%)	824/1353 (60.9%)	138/228 (60.5%)	2423/4201 (57.6%)
Regulatory sites in PhosphoSitePlus with at least two low-throughput references				
Rhesus macaque	2080/5192 (40.0%)	251/698 (35.9%)	9/12 (75.0%)	7/13 (53.8%)
Mouse	2106/5090 (41.3%)	233/816 (28.5%)	8/9 (88.8%)	0/0 (N/A)
Chicken	1362/3059 (44.5%)	848/2260 (37.5%)	70/263 (26.6%)	67/333 (20.1%)
Zebrafish	1181/2658 (44.4%)	1109/3111 (35.6%)	31/67 (46.2%)	26/79 (32.9%)
Fruit fly	310/641 (48.3%)	1580/3862 (40.9%)	65/175 (37.1%)	392/1237 (31.6%)
Nematode	161/355 (45.3%)	1464/3525 (41.5%)	60/177 (33.8%)	662/1858 (35.6%)
Yeast	18/47 (38.2%)	326/946 (34.4%)	79/180 (43.8%)	1924/4742 (40.5%)
Slime mold	61/133 (45.8%)	547/1353 (40.4%)	89/228 (39.0%)	1650/4201 (39.2%)

Each cell contains a value in the format X/Y, where Y is the total number of known human KPSPs in the category and X is the number of those having a known regulatory function. The corresponding percentage is indicated in parentheses. These values are shown for all phosphorylation sites listed as having regulatory roles in PhosphoSitePlus, as well as only those sites for which at least two papers used low-throughput assays to establish the regulatory role.

## Discussion

Many biochemical processes depend on functional interactions between distinct biomolecules. Kinases are no exception, as their biological contribution depends on their ability to phosphorylate a protein substrate. As such, protein phosphorylation is equally dependent on the presence of the protein kinase as well as the occurrence of the phosphorylation site within a target protein. Kinase substrates are distinguished from the rest of the proteome on the basis of phosphorylatable residues (typically Ser, Thr, or Tyr, although other residues such as His can sometimes be phosphorylated) contained within a kinase recognition motif.

Considerable effort has been expended in understanding the evolutionary relationships within the kinome and the phosphoproteome. The eukaryotic protein kinase superfamily is believed to have originated in prokaryotic protein signalling and to have evolved into the superfamily of kinases found in eukaryotes today [12]. Using genomic analysis, it has been possible to compare orthologous kinases amongst organisms to establish their functional and evolutionary relationships [13]. While there is less certainty of the evolutionary patterns in the phosphoproteome, there is emerging consensus on its complexity, including the possible existence of two different functional categories of phosphorylation that may be subject to unique evolutionary constraints [11, 14]. The data presented in this study suggest that, not surprisingly, KPSP conservation tends to decrease as evolutionary distance increases, and the absence of a particular KPSP is more likely to be due to the loss of a phosphorylation site rather than the loss of the protein kinase that phosphorylates it. We have provided a quantitative view of the extent of KPSP conservation at different evolutionary distances; specifically, Fig 3 shows that most human KPSPs are conserved in rhesus macaque, about half are conserved in chicken and zebrafish, and only a small proportion are conserved in distantly-related organisms like fruit fly, nematode, yeast, and slime mold. It is important to note that despite the quantitative view of kinase-phosphorylation site evolution presented here, these data are not yet enough to give statistically rigorous support to one model of KPSP establishment over the other. Such a statistical examination would be quite complex and thus outside the scope of this study; further, we are not confident that the data gathered here, which consists of predicted (rather than experimentally verified) conservation data, are sufficient to test this in a statistically rigorous way. Future studies that build on the work presented here could involve the development of statistical models for testing the kinase-first or phosphorylation site-first hypotheses, and would likely require the use of experimentally-verified rather than predicted conservation data.

Previous investigations have taken similar bioinformatics approaches to define the extent of evolutionary conservation of phosphorylation sites [11]. However, the priority of the current paper is distinct in offering a holistic perspective on the evolution of kinase-mediated phosphorylation by considering the extent of conservation of phosphorylation sites within the context of the modifying kinases. The central conclusions of this analysis are that the conservation of KPSPs depends on the evolutionary distance separating the species and that the vast majority of examples of incomplete KPSPs are due to the absence of the phosphorylation site. A number of arguments support the validity and biological significance of this conclusion.

While some studies have postulated strong evolutionary constraints on phosphorylation sites [15], other found only relatively weak constraints imposed on proteins by their phosphorylation [16, 17]. These differing conclusions may reflect two distinct types of protein phosphorylation. The first corresponds to modifications in which phosphorylation functions as a molecular switch to regulate protein function in a binary fashion [16, 18, 19]. These types of phosphorylation events tend to be more highly conserved [14]. Mutations to phosphorylation sites in this category typically have profound consequences affecting some critical functional aspect of the protein, such as activation, localization, stability, interacting partners, and so on [16, 18–21]. Such changes can have immediate and dramatic phenotypic consequences [22, 23]. The second category represents instances where the contribution of the phosphorylation event is through an aggregate action rather than binary regulation. In these instances, individual phosphorylation events are often a part of a cluster of phosphorylatable residues that undergo coordinated modification by a single kinase. In these instances, each phosphorylation event contributes to a more graduated influence on the protein’s function in a manner that is largely independent of the specific location of the phosphorylation site [14]. These types of phosphorylation sites do not tend to experience the same degree of evolutionary constraints as the first category. Indeed, “clusters” of phosphoacceptor sites may shift within the context of rapidly disordered regions of a protein to the generation of lineage-specific differences in phosphorylation-mediated regulation [24, 25].

Another explanation for the relatively poor degree of conservation of phosphorylation sites across species may reflect the biological insignificance of some of these phosphorylation events. Such insignificance could occur in two ways. First, the phosphorylation reaction may not occur *in vivo*, but rather represent an artefact of either the high throughput methodologies used to identify these modifications or non-specific interactions/modifications that occur under experimental conditions that do not reflect the cellular environment. Therefore, the phosphorylation site does not appear to be conserved because it is not actually biologically relevant. Secondly, biological insignificance may also be reflected in phosphorylation events that occur *in vivo*, but lack a biological function. Within the cellular environment, kinases, which often have low substrate specificity requirements, are presented with the opportunity for modification of a spectrum of proteins, some of which may result in inconsequential phosphorylation events [16, 26, 27]. Such non-functional phosphorylation events may offer an explanation for the lack of conservation of a phosphorylation site in orthologous proteins, in particular in closely related species. Given the low energy cost of phosphorylation, there would not be strong evolutionary pressure to eliminate such non-functional phosphorylation events. Further, while these individual phosphorylation events may not serve a specific biological function, random phosphorylation events of proteins may function as a critical evolutionary reservoir to fuel the emergence of new mechanisms of protein regulation and establishment of new signalling pathways.

In this study, we used only known KPSPs from human, a decision motivated by both simplicity of analysis and the fact that most known KPSPs are from human. However, this means that the study does not include any information on KPSPs from other organisms that are not conserved in human. Subsequent studies could involve the use of known KPSPs from all organisms from which they are available. Besides increasing the overall thoroughness of the analysis, this would provide a better balance between KPSPs in lower versus higher eukaryotes.

## Supporting information

S1 File DAPPLE hits against known phosphorylation sites in *Mus musculus* (mouse) and *Gallus gallus* (chicken). (XLSX)

S2 File Conservation of each known human KPSP with each of the eight organisms analyzed in this study. (XLSX)
